# A Simple, Robust, and Convenient HPLC Assay for Urinary Lactulose and Mannitol in the Dual Sugar Absorption Test

**DOI:** 10.3390/molecules27092677

**Published:** 2022-04-21

**Authors:** Ivana R. Sequeira, Marlena C. Kruger, Roger D. Hurst, Roger G. Lentle

**Affiliations:** 1 Human Nutrition Unit, School of Biological Sciences, University of Auckland, Auckland 1024, New Zealand; 2 School of Health Sciences, College of Health, Massey University, Palmerston North 4472, New Zealand; m.c.kruger@massey.ac.nz (M.C.K.); r.g.lentle@massey.ac.nz (R.G.L.); 3 Food Innovation Portfolio, The New Zealand Institute for Plant & Food Research Ltd., Palmerston North 4474, New Zealand; roger.hurst@plantandfood.co.nz

**Keywords:** high-performance liquid chromatography, intestinal permeability, lactulose, mannitol, dual sugar absorption test

## Abstract

Background: Heterogeneous laborious analytical methodologies for the determination of urinary lactulose and mannitol limit their utility in intestinal permeability testing. Methods: We developed an assay using a Shimadzu HPLC system, an Aminex HPX87C column, and refractive index detection. The test was calibrated using a series of dilutions from standard stock solutions of lactulose and mannitol ‘spiked’ into urine samples. The utility to quantify urinary excretion during the dual sugar absorption test over 6 h was also determined. Results: Lactulose and mannitol were eluted isocratically at 5.7 and 10.1 min, respectively, with water as a mobile phase at a flow rate of 0.3 mL min^−1^, 858 psi, 60 °C. The calibration curves for both sugars were linear up to 500 µg mL^−1^ with a limit of detection in standard solutions at 4 µg mL^−1^ and in ‘spiked’ urine samples at 15 µg mL^−1^. The intra-assay and inter-assay CVs were between 2.0–5.1% and 2.0–5.1% for lactulose and 2.5–4.4% and 2.8–3.9% for mannitol. The urinary profiles of the 6 h absorption of lactulose and mannitol showed similar peak-retention times to standard solutions and were well-resolved at 5.9 and 10.4 min, respectively. Conclusions: The assay was easy to automate, using commonly available equipment and convenient requiring no prior laborious sample derivatization. The simplicity, reproducibility, and robustness of this assay facilitates its use in routine clinical settings for the quantification of intestinal permeability.

## 1. Introduction

The standardization of a dual sugar absorption test using lactulose and mannitol [1] by our group with regard to the dosage and timing of urinary sample collection has enabled it to be used as a noninvasive quantitative assay for the direct assessment of small intestinal permeability. The lactulose mannitol (LM) test is useful for monitoring changes in intestinal permeability in a variety of conditions, such as celiac disease [2,3,4,5], Crohn’s disease [6,7,8], and food allergies [9,10,11], as well as in testing changes in permeability following the intake of aspirin [12,13] and ascorbic acid [14]. Lactulose and mannitol are useful probe sugars that are passively absorbed [15], nonmetabolized [16], water soluble, noncharged, nontoxic sugars that are quantitatively cleared by the kidneys in urine [16] within a six-hour period and can be readily quantified in urine [17]. Both lactulose and mannitol are chemically inert and uncharged molecules. The former is a disaccharide (molecular weight (MW): 342 Da; molecular radius: 0.42 nm) that is absorbed via the paracellular pathway [18], while the latter is a monosaccharide (MW: 182 Da; molecular radius: ≤0.4 nm) that is absorbed transcellularly across the luminal mucosa [18]. Despite the widespread utility and benefits of the LM test, heterogeneity in analytical protocols, including instrument performance and subsequent reporting, has limited its routine use [19] for measuring intestinal permeability.

Urinary lactulose and mannitol have been determined using various published methodologies that include enzymatic assays [20,21,22], colorimetric assays [23], and thin-layer chromatography (TLC) [24], as well as separate assays that individually quantify lactulose and mannitol [25,26]. These methods are not only technically laborious, but some also require the derivatization of urine samples prior to analysis. Recent work has indicated that high-performance liquid chromatography (HPLC) [27,28,29,30] may be a preferred methodology on the basis of its greater precision and sensitivity [31] in quantifying lactulose and mannitol [32,33,34,35]. However, the use of a number of different detectors and column types, mobile phases, flow rates [15,32,33,36,37], and sample preparation protocols has confound comparisons of their usefulness in clinical studies.

The use of different methods of detection stems from the fact that both lactulose and mannitol lack chromophore and flurophore groups [38]. Whilst the ultraviolet detection (UV) of an HPLC eluant with a photodiode array may give greater precision, this may require more complex and expensive equipment, such as mass spectrometry (MS) with electrospray ionization in tandem with MS [39]. Whilst MS is a highly sensitive and reliable method, its use has often involved a variety of detectors [15,32,33,36,37,40] that are expensive, which may limit its use during routine analysis in clinical settings [41].

Hitherto, considerations for method development have included an isocratic HPLC system with refractive index detection [27,28,30,40]. This work describes the development of a simple, reproducible, and convenient method for detecting urinary lactulose and mannitol based on HPLC methodology using a resin-based cation exchange column with a refractive index detector and a common, standard, readily available HPLC system. We determined the accuracy, sensitivity, precision, and reproducibility of the method using standard solutions, human urine samples spiked with lactulose and mannitol, and urine from subjects following the oral ingestion of a lactulose (10 g) and mannitol (5 g) solution during a standardized 6 h dual sugar absorption test.

## 2. Results

**Figure 1:**
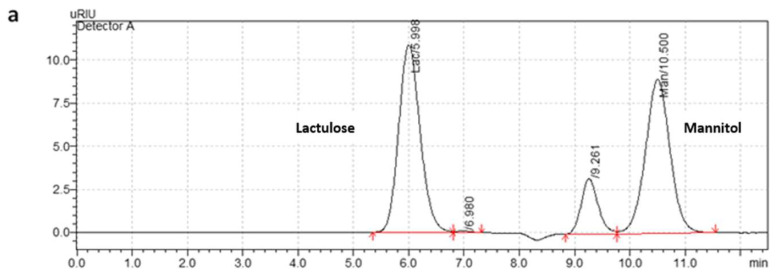

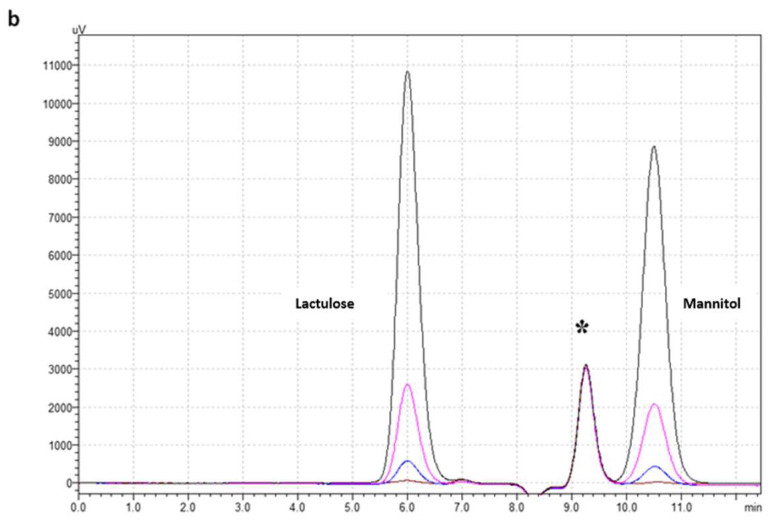
Chromatographic separation of lactulose and mannitol in standard solutions. Chromatograms depict (**a**) the resolution and peak separation of standard solutions containing 250 μg mL^−1^ lactulose and mannitol at 5.7 and 10.1 min, respectively. (**b**) Each sugar was eluted at the same retention time following injection of a range of different concentrations of standard solutions that contained 500 μg mL^−1^ (black), 125 μg mL^−1^ (pink), 31.3 μg mL^−1^ (blue), and 3.9 μg mL^−1^ (brown) lactulose and mannitol. A consistent residual peak before that of the mannitol peak from the diluent (water) is shown with an asterisk.

The chromatographic separation of lactulose and mannitol was achieved at a flow rate of 0.3 mL min^−1^. The analysis was performed at 60 °C, 858 psi, with a run time of 12 min for the standards and 25 min for urine samples (Figure 1a), with lactulose and mannitol eluting at 5.7 and 10.1 min, respectively. No major interference was seen at the retention times corresponding to the peaks at which each of the sugars were eluted. A relatively small, unknown peak with a constant area under the curve (AUC), which was due to a diluent contaminant, was eluted before mannitol (Figure 1b) and did not interfere with the mannitol peak resolution.

**Figure 2:**
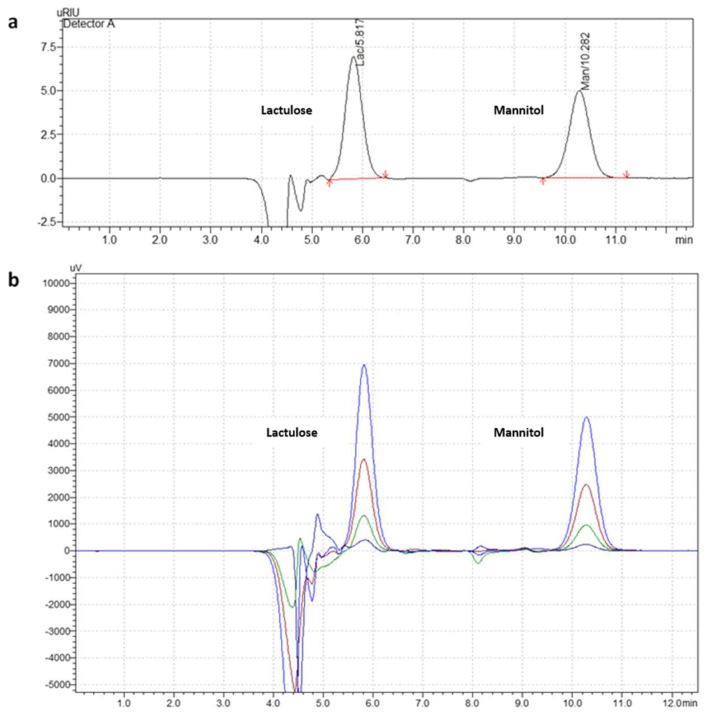
Chromatographic separation of lactulose and mannitol in urine samples spiked with varying concentrations of standard solutions. Chromatograms depict (**a**) the resolution and peak separation of urine spiked with standard solution containing 250 μg mL^−1^ lactulose and mannitol (corrected with the ‘blank’ un-spiked urine sample) at 5.8 and 10.2 min, respectively. (**b**) Each of the sugars eluted at the same retention time following injection of a range of different concentrations of standard solutions that contained 500 μg mL^−1^ (blue), 250 μg mL^−1^ (brown), 125 μg mL^−1^ (green), and 62.5 μg mL^−1^ (black) lactulose and mannitol after correction with the ‘blank’ un-spiked urine sample.

The chromatograms of the urine samples spiked with standard solutions containing lactulose and mannitol were well-resolved within 11 min (Figure 2a,b) with similar peak-retention times and resolutions as those obtained from the standards.

### 2.1. Calibration of Assay

**Figure 3:**
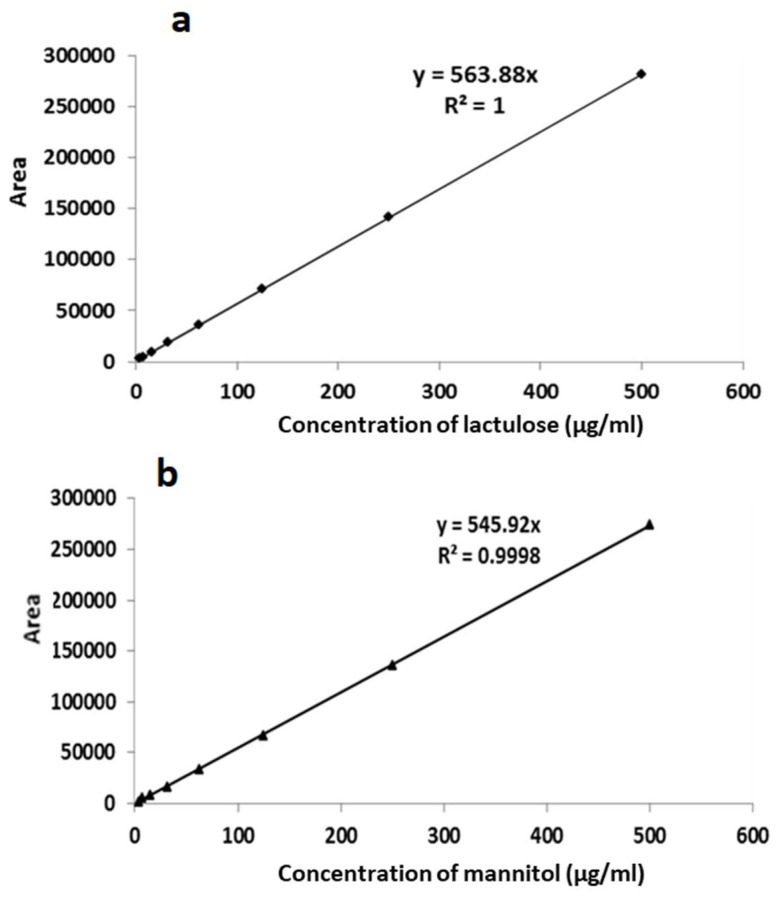
Linear calibration curves from standard solutions for (**a**) lactulose and (**b**) mannitol. The concentration of lactulose and mannitol in each standard was 500 μg mL^−1^, 250 μg mL^−1^, 125 μg mL^−1^, 62.5 μg mL^−1^, 31.3 μg mL^−1^, 15.6 μg mL^−1^, 7.8 μg mL^−1^, and 3.9 μg mL^−1^, respectively.

The calibration curves for lactulose (Figure 3a) and mannitol (Figure 3b) were each linear up to 500 µg mL^−1^ with the following regression equations: lactulose, y = 563.88x; and mannitol, y = 545.92x (expressed as the formula y = a + bx where a = 0 as the line intercepts the axes at 0). The limit of detection (LOD) was up to 4 µg mL^−1^ for both lactulose and mannitol in the standards.

### 2.2. Calibration with Spiked Urine Samples

The mean ratio of the observed-to-expected (O/E) recoveries for lactulose and mannitol for the set of spiked urine samples injected in triplicate over a range of dilutions ranged from 90–101% for both lactulose and mannitol across all the dilutions, indicating the sufficient linearity of the assay (Table 1).

The mean recovery for each of the sugars (to test the accuracy of the assay) from three different spiked urine samples ranged between 96% and 110% for lactulose and between 102% and 110% for mannitol (Table 2). Based on these recoveries of the sugars, the LOD for lactulose and mannitol from spiked urine samples was 15 µg mL^−1^.

### 2.3. Precision and Reproducibility

The precision of the assay for detecting lactulose and mannitol examined as a coefficient of variation (CV) for intra-assay variability was between 2.0% and 5.1% for lactulose and between 2.5% and 4.4% for mannitol (Table 3). 

The reproducibility of the assay for the detection of lactulose and mannitol, determined as an inter-assay CV, was between 2.0% and 5.1% for lactulose and between 2.8% and 3.9% for mannitol (Table 4).

### 2.4. Detection of LM in Urine Samples during the Dual Sugar Absorption Test

**Figure 4:**
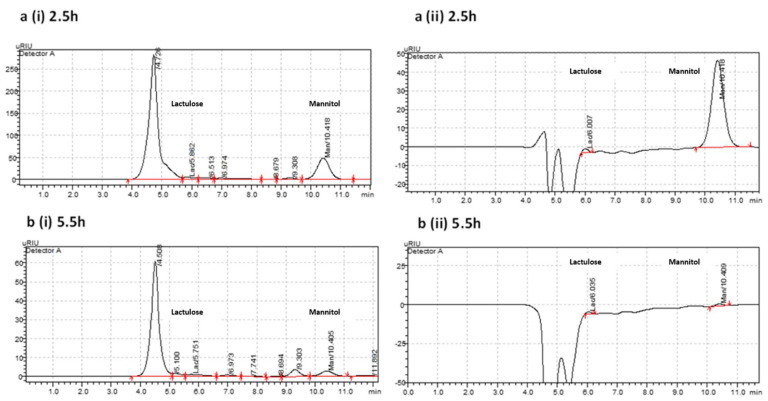
Detection of lactulose and mannitol over a 6 h testing period. Lactulose and mannitol were well-resolved in urine samples at 5.9 and 10.4 min, respectively. Chromatograms depict peaks of each sugar in urine samples before (**a**(**i**),**b**(**i**)) and after (**a**(**ii**),**b**(**ii**)) correction of the respective sample with the baseline ‘blank’ urine sample at 2.5 h and 5.5 h post-ingestion of the lactulose mannitol test solution.

The sensitivity and reproducibility of the methodology for detecting each sugar in urine samples during the dual sugar absorption test for intestinal permeability was also tested and showed good peak resolution of lactulose and mannitol at 5.9 and 10.4 min, respectively, in all samples collected over a 6 h period of testing. The peaks were distinct and well-defined in samples collected at the start (Figure 4a(i)), as well as toward the end (Figure 4b(i)), of the testing period. The correction of the chromatograms obtained from half-hourly urine samples with that obtained from the baseline ‘blank’ sample showed a clear AUC with distinct peak separation and resolution (Figure 4a(ii),b(ii)).

## 3. Discussion

The HPLC assay that was developed in our laboratory proved to be simple to use, robust, and reproducible. As such, it can readily be used in hospital laboratories to detect trace quantities of lactulose and mannitol to assess small intestinal permeability. The methodology involved no equipment in addition to that which is normally used in clinical laboratories and was easy to automate. In particular, it required no prior sample derivatization or internal standard, as has been reported in previous published methodologies. The simplified and convenient assay had similar CVs for precision and reproducibility to other reported studies based on urine samples to assay intestinal permeability [27,42].

The chromatographic resolution and separation were well-defined over the entire 6 h period of testing and allowed for the temporal patterns of the excretion of lactulose and mannitol to be accurately determined [12,13] and the LM ratio to be derived, as it is often used as a cutoff for defining increased intestinal permeability. While a small peak was eluted just prior to the peak in mannitol, comparison and assessment of the constancy of the peak to that obtained from a pure ‘diluent’ solution (i.e., filtered MilliQ) showed that it was due to the presence of an unknown substance in the diluent. Again, the retention time of this peak was sufficiently different from that of mannitol (9.08 min) to allow the AUC of mannitol to be well-separated and accurately quantified, and had no impact on the assay.

In prior publications based on a series of randomized crossover human studies [12,13,14,43], we have standardized the clinical testing methodology with regard to the proper dosage and timing of urine sample collection [1] for assessing region-specific intestinal permeability. Additionally, the methodology and protocol adopted for the dual sugar absorption test allowed the temporal patterns of absorption of the individual sugars to be compared before and after a single dose of aspirin. This provided the opportunity to acquire information on the differences in digestibility and the degradation of the component sugars within the different segments of the gut. The consistency of the results obtained from these clinical studies using the developed HPLC method further support repeatability and reproducibility in diagnostic clinical settings [12,13,14,43].

Endogenous mannitol is known to be naturally present in urine in low concentrations. Trace amounts of mannitol in dietary items that are consumed during the 24 h prior to testing, along with traces from endogenous production by commensal intestinal microflora [44], necessitate the correction of samples by subtraction of the quantities found in a baseline sample collected prior to the administration of the LM solution [29,45]. Note that this procedure assumes a constancy of excretion during the period of the test. An additional strength of our method is that the baseline levels can be determined with sufficient accuracy to allow meaningful correction.

The methodological modifications incorporated during the development of the assay included the use of a resin-based column, Aminex HPXC, which exhibits multimodal interactions described as ion-moderated partitioning [46]. The use of this resin is advantageous over other reversed-phase and ion-pairing methodologies, which work on the principle of modifying the compound to be analyzed and require complex eluants for effective separation. This feature enabled an isocratic HPLC system to be used and the method of sample preparation to be simplified, avoiding the need for the derivatization of the sample. The bonded resin of the columns are prone to retaining certain components of the sample that progressively decrease its efficiency and selectivity [47]. This problem was successfully avoided by the use of a Micro-Guard pre-column that was installed between the injector and the analytical column to protect from plugging and contamination by samples and the mobile phase. A limitation of the use of refractive index detection is its susceptibility to baseline drifts that may result from temperature and pressure fluctuations. This was overcome by the placement of the detector within the HPLC system away from any vents, and the detector was warmed for at least two hours prior to sample injection.

In conclusion, the described methodology was simple, readily automated, convenient, reproducible, and robust for use in routine clinical settings and facilitated the ease of undertaking an LM test over a six-hour period. Intestinal permeability is recognized as an important diagnostic marker, commonly measured using the dual sugar absorption test [48] and is a highly useful tool for screening gut ‘leakiness’. Although there is a need for accurately assessing intestinal permeability, the multiplicity of current methods limits its utility [49]. It is anticipated that the use of a standardized LM testing protocol with regard to the dosage and timing of urine sample collection in conjunction with the methods described herein may facilitate routine intestinal permeability testing within clinical settings.

## 4. Materials and Methods

*Chemicals and Reagents:* Lactulose (L7877-25G) and mannitol (M4125-500G) were purchased from Sigma Aldrich, Saint Louis, MO, USA. The Amberlite resins IR120 H and IRA410 Cl were obtained from Fluka, Sigma Aldrich, Saint Louis, MO, USA. Cellulose acetate filters (pore size of 0.2 µm) were supplied by Advantec, Toyo Roshi Kaisha Ltd., Tokyo, Japan. HPLC vials (1.5 mL) were obtained from Fisher Scientific, Loughborough, LEI, UK. Filtered MilliQ water was utilized in all sample preparation. The urine samples for spiking and calibration were provided by one of the authors (IRS), following a routine 8–10 h overnight fast. The urine samples were aliquoted and stored at −80 °C pending spiking and HPLC analysis. The urinary sample aliquots were thawed at room temperature pending spiking with calibrators.

The stock solutions of both lactulose and mannitol were prepared in filtered MilliQ water and stored at 4 °C for one week. A series of dilutions containing the appropriate aliquots of each of the two standard stock solutions in filtered MilliQ water was prepared (see below). Similarly, the serial dilutions of each of the two standard stock solutions in human urine were prepared using filtered MilliQ water. Each of the series of dilutions was then stored at 4 °C for one week.

*Sample preparation and treatment:* A standard stock solution (1 mg mL^−1^) was prepared using 100 mg lactulose (Sigma Aldrich L7877-25G) and 100 mg mannitol (Sigma Aldrich M4125-500G) dissolved in and made up to 100 mL of filtered MilliQ water at room temperature. A series of dilutions from 500 µg mL^−1^ to 3.9 µg mL^−1^ was prepared from the above stock solution.

An amount of 1 mL of urine sample was spiked with 1 mL of the requisite standard to obtain the required final concentration for calibration (Table 5). Each urine sample was then treated with 500 mg Amberlite IR120 H and 500 mg of Amberlite IRA410 Cl.

Prior to injection on the HPLC, the standards and spiked urine samples were vortex-mixed for 15 s and filtered through a 0.2 µm cellulose acetate filter into 1.5 mL HPLC vials.

*Chromatographic conditions:* The samples were chromatographed on a Shimadzu HPLC system equipped with a DGU-20AS Degasser Model (Kyoto, Japan), an LC-20AT Prominence Pump Model, an SIL-20AC Autosampler Model, and a CTO-20A Column Oven using an Aminex HPX87C 250 × 4.0-mm column and a Micro-Guard Cartridge pre-column. LM were eluted isocratically with water as the mobile phase at a flow rate of 0.3 mL min^−1^, 858 psi, at 60 °C. The injection volume was 20 µL, and the run time was set to twenty-two min for the elution of urine samples. The chromatograms obtained from urine samples spiked with the standards were corrected with a ‘blank’ unspiked urine sample using LC solutions software in the post-run mode. The AUC of each of the sugars in each urine sample was then determined and compared to those of a series of standards run concurrently using LC solutions software.

*Method validation:* The LOD, linearity, precision, and accuracy of the assay were determined.

*Calibration curves with reference solutions:* The detector response was assessed using different concentrations of standard lactulose and mannitol solutions (500 µg mL^−1^, 250 µg mL^−1^, 125 µg mL^−1^, 62.5 µg mL^−1^, 31.3 µg mL^−1^, 15.6 µg mL^−1^, 7.8 µg mL^−1^, and 3.9 µg mL^−1^) run in triplicate, and the LOD was determined.

*Calibration curves with spiked urine samples:* 1 mL of urine sample was spiked with 1 mL of different standards to obtain the final concentrations of 500 µg mL^−1^, 250 µg mL^−1^, 125 µg mL^−1^, 62.5 µg mL^−1^, and 31.3 µg mL^−1^ (Table 5) and run in triplicate, and the LOD was determined.

*Linearity of the assay:* The dilutional linearity, or the recovery of LM from the samples, was used to validate the specificity and accuracy of the methodology. The urine samples were spiked with standards containing lactulose and mannitol to obtain concentrations of 250 µg mL^−1^, 190 µg mL^−1^, and 125 µg mL^−1^ and diluted in 1:2, 1:4, and 1:8 proportions with filtered MilliQ water. Each dilution was run in triplicate, and the results were expressed as observed in the O/E ratios of recovery for each sugar.

*Accuracy of the assay:* The accuracy of the assay expressed the degree of closeness between the reference values, i.e., the concentration in the standard, to that obtained from the found value, i.e., in the spiked sample. This was determined by evaluating the recovery of three different concentrations of urine samples spiked with standard solutions and results expressed as O/E ratios. Three urine samples having final concentrations of 250 µg mL^−1^, 62.5 µg mL^−1^, and 15.6 µg mL^−1^ of both lactulose and mannitol were injected in 10 separate aliquots (repeated 10 times for each concentration within the same run) to allow the mean, standard deviation (SD), CV, and standard error (SE) for each sugar to be calculated.

*Precision of the assay:* The precision of the assay expressed the closeness of a series of individual measurements when the assay was applied repeatedly to multiple aliquots of a single homogenous volume of the sample. This was determined by evaluating the intra-assay variability by measuring three different concentrations, 500 µg mL^−1^, 125 µg mL^−1^, and 31.3 µg mL^−1^, of lactulose and mannitol in spiked urine samples injected 10 times within the same run, and the mean, SD, CV, and SE for each sugar were calculated.

*Reproducibility of the assay:* The reproducibility of the assay, or the repeatability of the assay, was determined by evaluating inter-assay variability by measuring three different concentrations of spiked urine samples injected in consecutive assay runs, and the mean, SD, CV, and SE for each sugar were calculated. Urine samples having final concentrations of 250 µg mL^−1^, 125 µg mL^−1^, and 31.3 µg mL^−1^ of lactulose and mannitol were injected in 10 consecutive assay runs.

*Utility of the method to detect lactulose and mannitol in urine samples collected during routine dual sugar absorption test:* Ethics approval was obtained from the Massey University Human Ethics Committee Southern A: 09/79 [12], 11/37 [13], 12/13 [14], 13/31 [43]. All participants provided written informed consent prior to the assessments. Briefly, following a standard 8–10 h overnight fast, healthy female participants attended the Human Nutrition Laboratory, Massey University, Palmerston North Campus. They refrained from consuming prebiotic and probiotic supplements, such as lactulose, and nonsteroidal anti-inflammatory drugs for one week prior to the test, from consuming alcohol for three days prior to the test, and avoided exercise on the day before and the morning of the test. A baseline urine sample was collected on arrival at the laboratory. The participants consumed a solution containing 10 g lactulose (Duphalac, Solvay Pharmaceuticals, Sydney, NSW, Australia) and 5 g D-mannitol (Sigma Aldrich, Saint Louis, MO, USA). Half-hourly urine samples were collected following the ingestion of the LM solution over a 6 h period. No food was consumed during the entire urine collection period, but 200 mL of water was given three hours after the ingestion of the sugar solution to facilitate urine sample collection.

The volume of each of the half-hourly urine samples was recorded. They were then centrifuged (Heraeus Sepatech Megafuge 1.0R) at 4500 rpm (3500× *g*) at 14 °C for 10 min immediately following collection. An amount of 20 mL of supernatant was aliquoted and stored at −80 °C until it was thawed at room temperature for HPLC analysis. All urine samples were treated with a 1:1 proportion of amberlite resin (500 mg Amberlite IR120 H and 500 mg of Amberlite IRA410 Cl), vortex-mixed for 15 s, and then filtered using 0.2 µm cellulose acetate filters into HPLC vials. A 20 μL aliquot of filtrate from each sample was then injected into an HPLC system. The chromatograms obtained were corrected with the baseline ‘blank’ using LC solutions software in the post-run mode.

## Figures and Tables

**Table 1:** Ratio of the observed-to-expected (O/E) recoveries of lactulose and mannitol using three spiked urine samples.

O/E Ratio (%)	1:2		1:4		1:8	
	Lac	Man	Lac	Man	Lac	Man
Mean ^a^	100.6 ± 6.8	97.7 ± 1.7	93.9 ± 4.2	89.8 ± 4.0	89.5 ± 7.9	93.7 ± 8
SE	0.87	0.44	0.69	0.67	0.94	0.94
CV%	6.8	1.8	4.5	4.5	8.9	8.6
Min	90	95	91	84	81	81
Max	113	100	105	97	100	102

^a^ Data in this row are presented as mean ± standard deviation of the O/E ratio in each of the dilutions as a composite for both the sugars in the three different urine samples (total data points: 27). Urine samples were spiked with standards and had final concentrations of 250 µg mL^−1^, 190 µg mL^−1^, and 125 µg mL^−1^, which were used in 1:2, 1:4, and 1:8 dilutions. Min and max are values of percentage recoveries. SE = standard error; CV% = coefficient of variation.

**Table 2:** Recovery of lactulose and mannitol from three different spiked urine samples.

O/E Ratio (%)	Sugar Added ^a^					
	Sample 1		Sample 2		Sample 3	
	Lac	Man	Lac	Man	Lac	Man
Mean ^b^	108.9 ± 3.2	110.3 ± 2.8	95.8 ± 2.0	101.8 ± 4.4	103.2 ± 5.3	102.5 ± 10.5
Min	105	106	93	97	95	92
Max	113	116	99	111	113	130

^a^ Lactulose + mannitol; ^b^ data are expressed as mean ± standard deviation of the O/E ratio for both sugars in the three different urine samples (total data points: 30). Urines were spiked with standards and had final concentrations of: Sample 1 = 250 µg mL^−1^, Sample 2 = 62.5 µg mL^−1^, and Sample 3 = 15.6 µg mL^−1^. Min and max are values of percentage recoveries.

**Table 3:** Intra-assay variability of lactulose and mannitol in three urine samples each repeated 10 times within the same assay run.

Urine Spike	Sample 1		Sample 2		Sample 3	
	Mean ^a^ (µg mL^−1^)	CV ^b^ (%)	Mean ^a^ (µg mL^−1^)	CV ^b^ (%)	Mean ^a^ (µg mL^−1^)	CV ^b^ (%)
Lactulose	544.4 ± 15.9	2.9	119.7 ± 2.5	2.1	32.2 ± 1.6	5.1
Mannitol	551.7 ± 13.9	2.5	127.3 ± 5.5	4.3	31.1 ± 1.4	4.4

^a^ Mean ± standard deviation of the concentration of sugar in urine sample from the 10 measurements within the same run; ^b^ the coefficients of variation for each sugar. Urine samples were spiked with standards and had final concentrations of: Sample 1 = 500 µg mL^−1^, Sample 2 = 125 µg mL^−1^, and Sample 3 = 31.3 µg mL^−1^.

**Table 4:** Inter-assay variability of lactulose and mannitol in three urine samples each repeated 10 times in consecutive assay runs.

Urine Spike	Sample 1		Sample 2		Sample 3	
	Mean ^a^ (µg mL^−1^)	CV ^b^ (%)	Mean ^a^ (µg mL^−1^)	CV ^b^ (%)	Mean ^a^ (µg mL^−1^)	CV ^b^ (%)
Lactulose	251.0 ± 5.0	2.0	126.4 ± 4.0	3.1	31.6 ± 1.6	5.1
Mannitol	245.8 ± 6.8	2.8	121.4 ± 4.5	3.7	30.4 ± 1.2	3.9

^a^ Mean ± standard deviation of the concentration of sugar in urine samples from the 10 consecutive assay runs; ^b^ the coefficients of variation for each sugar. Urine samples were spiked with standards and had final concentrations of: Sample 1 = 250 µg mL^−1^, Sample 2 = 125 µg mL^−1^, and Sample 3 = 31.3 µg mL^−1^.

**Table 5:** Dilutions used for preparation of spiked urine samples for calibration curves.

Initial Std conc (µg mL^−1^)	1000	500	250	125	62.5	313.3	15.6	7.8
Std (mL)	1	1	1	1	1	1	1	1
Urine (mL)	1	1	1	1	1	1	1	1
Final ‘spiked’ urine sample conc (µg mL^−1^)	500.0	250.0	125.0	62.5.0	31.3	15.6	7.8	3.9

Std: standard; conc: concentration.

## Data Availability

De-identified data will be shared and made available upon reasonable request to the corresponding author subject to an approved proposal and data access agreement.

## Sample Availability

Samples of the compounds are not available from the authors.
